# Comment on: Month of birth and risk of multiple sclerosis: confounding and adjustments

**DOI:** 10.1002/acn3.53

**Published:** 2014-04-10

**Authors:** Barnaby Fiddes, James Wason, Stephen Sawcer

**Affiliations:** 1 Department of Clinical Neurosciences, University of CambridgeBox 165, Cambridge Biomedical Campus, Hills Road, Cambridge, CB2 0QQ, United Kingdom; 2 Medical Research Council Biostatistics UnitCambridge, CB2 0SR, United Kingdom

Dear Editor,

We read the latest paper from Torkildsen et al.^1^ with great interest and agree with the authors that their data provide a convincing empirical demonstration of the extent to which failing to correct for year and place of birth can generate false-positive associations in studies considering the role of month of birth (MOB) in multiple sclerosis.^2^ However, regarding their suggestion that these data might still provide evidence in support of a MOB effect in the disease, we would make three observations. First, while we accept that this particular set of Norwegian cases has a high number of April births, we do not see why this would be considered as evidence for a MOB effect when the authors show that after Bonferroni correction the observed excess is not statistically significant. Indeed after Bonferroni correction, there are no statistically significant differences between the cases and any of the listed control cohorts – population, siblings, or parents. Given the minimal correlation in a multinomial distribution with 12 outcomes, a Bonferroni correction seems appropriate rather than overly conservative. The authors' suggestion that this nonsignificant trend might still be considered as evidence of a genuine effect, brings us to our second point. Has their correction for place of birth been adequate, does correction to the level of counties provide sufficient matching? Analysis of population statistics from U.K. local authorities shows that heterogeneity in MOB is still evident in populations of circa 200,000 indicating that residual heterogeneity would be expected at the level of Norwegian counties (average population 262,414 = 4,985,870/19).^2^ It, therefore, seems likely that to fully exclude confounding due to place the authors might need to match to the level of municipalities and not just counties. In short, the trend towards excess births in April observed by the authors seems more likely to have resulted from inadequate compensation for place of birth, rather than any real effect. Finally, although the authors' study is detailed it has very little power, making it further unlikely that any observed trends represent a real effect. If MOB plays a role in multiple sclerosis its effect is modest, and expected to require the consideration of many tens of thousands of cases.^3^

To our minds, this new more detailed analysis of these existing data provides a compelling illustration of the effects of confounding but do not provide any convincing evidence for an underlying MOB effect in multiple sclerosis.
